# Acute effects of systemic inflammation upon the neuro-glial-vascular unit and cerebrovascular function

**DOI:** 10.1016/j.bbih.2020.100074

**Published:** 2020-04-22

**Authors:** Gaia Brezzo, Julie Simpson, Kamar E. Ameen-Ali, Jason Berwick, Chris Martin

**Affiliations:** a The University of Sheffield, Department of Psychology, Cathedral Court, 1 Vicar Lane, Sheffield, S1 2LT, UK; b The University of Sheffield, Sheffield Institute for Translational Neuroscience (SITraN), 385a Glossop Road, Sheffield, S10 2HQ, UK

**Keywords:** Inflammation, Lipopolysaccharide, Neurovascular coupling, Neuro-glial-vascular unit

## Abstract

Brain health relies on a tightly regulated system known as neurovascular coupling whereby the cellular constituents of the neuro-glial-vascular unit (NGVU) regulate cerebral haemodynamics in accordance with brain metabolic demand. Disruption of neurovascular coupling impairs brain health and is associated with the development of a number for neurological conditions, including Alzheimer’s disease. The NGVU is also a key site of action for neuroinflammatory responses and contributes to the transition of systemic inflammation to neuroinflammatory processes. Thus, systemic inflammatory challenges may cause a shift in NGVU operation towards prioritising neuroinflammatory action and thus altering neurovascular coupling and resultant cerebrovascular changes. To investigate this, rats were injected with lipopolysaccharide (LPS) (2 ​mg/kg) to induce a systemic inflammatory response, or vehicle, and brain haemodynamic responses to sensory and non-sensory (hypercapnia) stimuli were assessed *in vivo* using optical imaging techniques. Following imaging, animals were perfused and their brains extracted to histologically characterise components of the NGVU to determine the association between underlying cellular changes and *in vivo* blood flow regulation*.* LPS-treated animals showed changes in haemodynamic function and cerebrovascular dynamics 6 ​hours after LPS administration. Histological assessment identified a significant increase in astrogliosis, microgliosis and endothelial activation in LPS-treated animals. Our data shows that an acutely induced systemic inflammatory response is able to rapidly alter *invivo* haemodynamic function and is associated with significant changes in the cellular constituents of the NGVU. We suggest that these effects are initially mediated by endothelial cells, which are directly exposed to the circulating inflammatory stimulus and have been implicated in regulating functional hyperaemia.

## Highlights

• Systemic LPS treatment acutely alters cerebrovascular/imaging responses to whisker stimulation.
• Changes to functional hyperaemia are associated with altered neuro-glial-vascular unit status.
• LPS treatment induced rapid astrogliosis, microgliosis and endothelial cell activation.
• These data have implications for functional neuroimaging under systemic inflammatory conditions.

## 1 Background

Brain health and function are dependent on a comprehensively regulated blood supply. The local regulation of blood flow and cerebrovascular function in accordance with brain metabolic demand is termed neurovascular coupling and is orchestrated by the resident cells of the neuro-glial-vascular unit (NGVU). Disruption to neurovascular coupling can impair the delivery of critical substrates to brain cells and impede the removal of by-products accumulated during cerebral metabolism (Girouard and Iadecola, 2006). Alterations of brain microenvironment and cellular interactions of the NGVU have been implicated in the development of a number of neurodegenerative diseases, including Alzheimer’s disease (AD) (De Strooper and Karran, 2016; Sweeney et al., 2015; Zlokovic, 2011). Nevertheless the process or processes by which neurovascular coupling affects, and is affected by, neurodegenerative diseases *in vivo,* as well as the cellular substrates of these effects, remain unclear.

Neurovascular coupling underpins the physiological basis of non-invasive functional neuroimaging techniques, including functional magnetic resonance imaging (fMRI) positron emission tomography (PET) and infra-red spectroscopy (NIRS) in which changes to brain blood flow and oxygenation are tracked as surrogate markers for neuronal activity. Such neuroimaging techniques may provide new opportunities to predict, detect, diagnose and study brain disease processes using non-invasive imaging biomarkers. However, these possibilities are dependent on our understanding of the mapping of *in vivo* functional imaging measurements to neuropathological changes and this may itself be affected by specific disease processes such as inflammation.

Mounting evidence highlights inflammation as a major factor in the development of many neurodegenerative diseases (Cunningham, 2013; Frank-Cannon et al., 2009; Gao et al., 2011; Heppner et al., 2015). Further evidence pinpoints inflammation as a driver of neuropathology (Krstic et al., 2012) and it has been shown to precede the development of amyloid-beta (Aβ) plaques (Wright et al., 2013). The NGVU is the site of action of neuroinflammatory responses and contributes to the transition of systemic inflammation to neuroinflammatory processes. Several non-neuronal cells within the NGVU are key players in the initiation and regulation of brain inflammatory responses, as well as in mediating the effects of systemic inflammation upon brain function. Activated astrocytes and microglia release a range of pro-inflammatory molecules (Saijo et al., 2009; Boche et al., 2013; Sofroniew, 2009; Sofroniew and Vinters, 2010). Endothelial cells (ECs) also play an important role through upregulation of intercellular adhesion molecules (ICAM-1) and vascular cellular adhesion molecules (VCAM-1) (Huber et al., 2006). Research has also highlighted a beneficial role for inflammation, suggesting that activating the inflammatory response may be of more therapeutic benefit than suppressing it (Buckwalter and Wyss-Coray, 2004; Wyss-Coray and Mucke, 2002). Glial cells have been shown to have a neuroprotective role in the neuroinflammatory response (Morgan et al., 2004; Polazzi et al., 2001; Bush et al., 1999; Chen et al., 2001), highlighting the complexity and difficulty in pinpointing the roles and factors involved in the pathophysiological cascade of inflammation.

To help elucidate the changes that occur in the context of inflammation, numerous models have been developed. The peripheral lipopolysaccharide (LPS) injection method is a standard technique of inducing inflammation both *in vivo* (Hauss-Wegrzyniak et al., 1998; Pintado et al., 2012) and *in vitro* (Lehnardt et al., 2003). Depending on dosage, LPS treated animals display behavioural as well as cellular brain changes, predominately associated with glial activation (Nazem et al., 2015; Zakaria et al., 2017). The current study investigated how acute systemic inflammation impacts upon *in vivo* cerebrovascular function and the status of the underlying NGVU cells. This was investigated with a complementary set of *in vivo* neuroimaging measures in a rat model, paired with detailed characterisation of the cellular pathology of the NGVU in the same animals using immunohistochemistry methods.

## 2 Methods

The present study was approved by the UK Home Office under the Animals (Scientific Procedures) Act 1986 and the University of Sheffield Animal Welfare and Ethical Review Body (AWERB, local ethics committee). All procedures were conducted under a U.K. Home office licence and have been reported in accordance with the ARRIVE guidelines.

### 2.1 Animals and pharmacological treatment

**Fig. 1:**
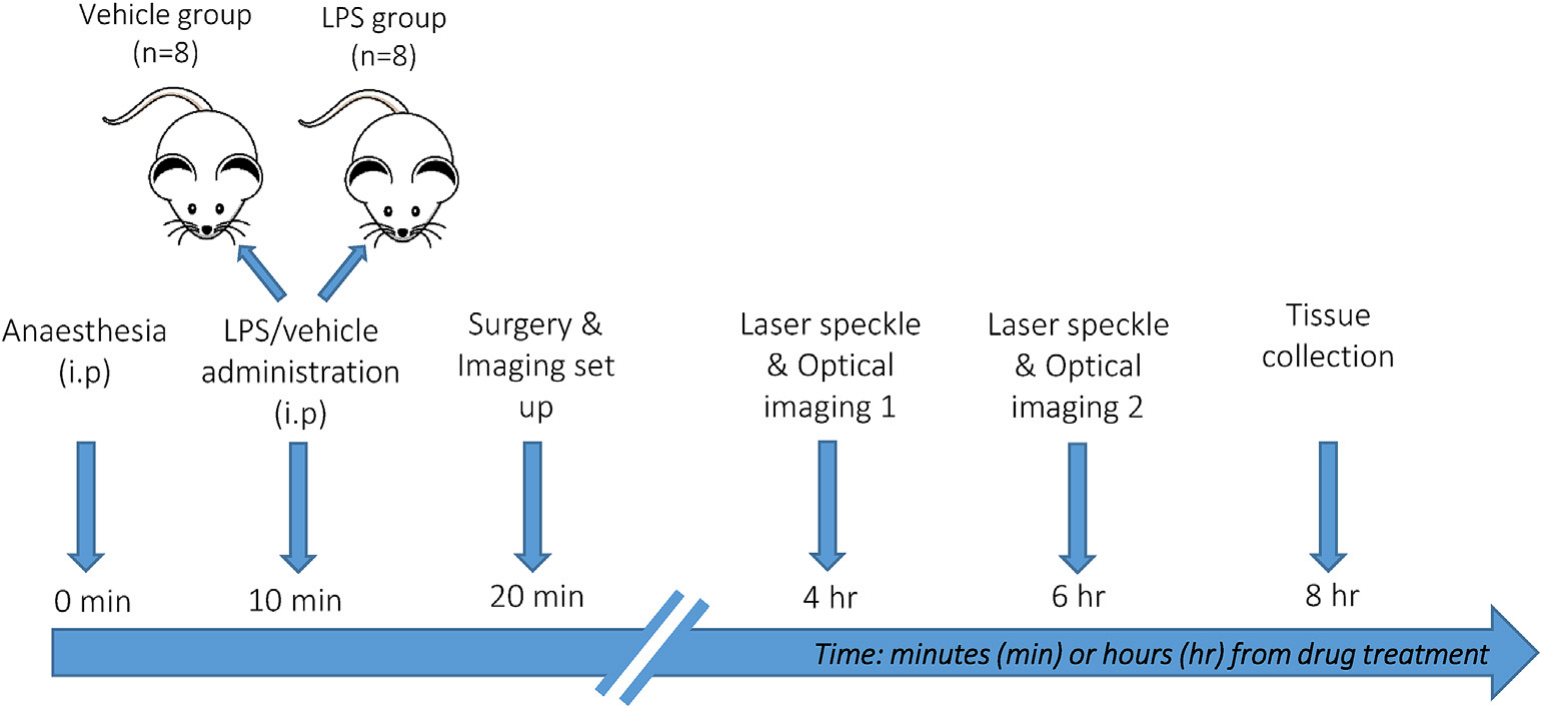
**Experimental timeline.** Graphical representation of study design and timeline from non-recovery general anaesthesia to tissue collection. Time (minutes or hours) is given from time of injection (0 ​min).

Female Hooded Lister rats (3–4 months old, 220g–320g) kept at a 12-h light/dark cycle environment at a temperature of 22 ​°C with access to food and water *ad libitum* were housed in polycarbonate cages (*n* ​= ​3 per cage) in the Biological Services Unit at the University of Sheffield*.* Animals were fed conventional laboratory rat food. Sixteen animals were randomly assigned to one of two groups (control *n* ​= ​8 or LPS, *n* ​= ​8). Haemodynamic data were acquired in all treatment groups at both 4 and 6 ​h after LPS/vehicle administration, to characterise any effects of the acutely induced LPS inflammatory response (Fig. 1).

Each animal received an intraperitoneal injection based on condition. Control animals were administered a saline vehicle (1 ​ml/kg), LPS animals received a dose of 2 ​mg/kg LPS-EB (lipopolysaccharide from E.coli, 0111:B4 stain-TLR4 ligand, InvivoGen, Europe) dissolved in endotoxin-free water (InvivoGen, Europe), following loss of consciousness from anaesthesia.

### 2.2 Surgical procedures

Details of surgical and experimental paradigms were similar to those reported in previous publications from this laboratory (Hewson-Stoate et al., 2005; Jones et al., 2001; Martindale et al., 2003). Briefly, rats were anaesthetised with an intraperitoneal injection (i.p.) of urethane (1.25 ​mg/kg in 25% solution), with additional doses of anaesthetic (0.1 ​ml) administered if necessary. Choice of anaesthetic was determined by urethane’s suitability for invasive surgery as well as long-lasting stability, which is essential in experiments where data collection lasts several hours (Angel and Gratton, 1982). Anaesthetic depth was initially determined by means of hindpaw pinch and corneal reflex testing. During data acquisition continuous blood pressure monitoring did not reveal physiological responses to our whisker stimulation protocol, which would have been indicative of inadequate anaesthetic depth. Animals were tracheotomised to allow artificial ventilation and regulation of respiratory parameters. A left femoral artery cannulation was performed for mean arterial blood pressure (MABP) monitoring and blood gas analysis. A left femoral vein cannulation was also performed to allow continuous administration of phenylephrine in order to maintain blood pressure within a healthy physiological range (100–110 ​mmHg) (Golanov et al., 1994).

To enable haemodynamic recordings, the skull was exposed via a midline incision and a section overlying the left somatosensory cortex (barrel cortex) was thinned to translucency with a dental drill. This section was located 1–4 ​mm posterior and 4–8 ​mm lateral to Bregma (Chapin and Lin, 1984). A thinned skull was typically 100–200 ​μm thick with the cortical surface vasculature clearly observable. Care was taken during thinning to ensure that the skull remained cool by frequently bathing the area with saline.

### 2.3 Physiological monitoring

Temperature was maintained at 37 ​°C (±0.5 ​°C) throughout surgical and data collection procedures with the use of a homoeothermic blanket and rectal temperature probe (Harvard Apparatus, USA). Animals were artificially ventilated with room air using a small animal ventilator (SAR 830, CWE Inc, USA); the breathing rate of each animal was assessed and modified according to each individual animal’s blood gas measurements. Respiration rates of the animals ranged from 68 to 74 breaths per minute.

Blood pressure was monitored during the experiment with a pressure transducer (Wockhardt, UK, 50 units of heparin per mL). Arterial blood from the femoral artery was allowed to flow back from the cannula into a cartridge (iSTAT CG4+, Abbott Point of Care Inc., USA) and blood gases were analysed to ensure normoxia and normocapnia using a blood gas analyser (VetScan, iSTAT-1, Abaxis, USA). Physiological parameters were within normal ranges throughout the experiment (mean values: PO₂ ​= ​80 ​mmHg(±9.1) PCO₂ ​= ​30.4 ​mmHg(±3.7) SO₂ ​= ​96%(±1.2)). Total volume of arterial blood extracted at one time did not surpass 95 ​μL. Phenylephrine (Sigma, Aldrich) was administered into the left femoral vein using a syringe pump (Sp200i, World Precision Instruments Inc., USA) to counteract reduced blood pressure caused by anaesthesia. The dose was adjusted according to the blood pressure of each individual animal but remained in the range of 0.6–2.0 ​mg/hr.

### 2.4 Imaging

Cerebral blood flow (CBF) data were acquired with a laser speckle contrast imaging (LSCI) camera (Full field Laser Perfusion Imager (FLPI-2), Moor Instruments, UK) which was positioned above the thinned cranial window. Images were acquired at 25Hz with a spatial resolution of approximately 10μm/pixel. A 70s baseline data acquisition was acquired to obtain a measure of baseline blood flow. A 3-D dataset comprising 2-D images of CBF changes over time was written to a computer hard-disk drive by proprietary software (Moor Instruments, UK).

The two-dimensional optical imaging spectroscopy (2D-OIS) technique was used to estimate activity-induced changes in oxygenated (HbO_2_), deoxygenated (HbR) and total (HbT) haemoglobin concentration in the rat barrel cortex. This technique has been previously described in detail (Berwick et al., 2005; Sharp et al., 2015). 2D-OIS data were collected at a frame rate of 8Hz and written to a computer hard-disk drive using software of in-house design. Subsequent off-line spectral analysis, conducted using MatLab, was based on the path length scaling algorithm (PLSA) (Berwick et al., 2005), which uses a modified Beer-Lambert Law with a path length correction factor. In our analysis, baseline haemoglobin concentration in the tissue was estimated to be 104 ​μm based on previous measurements (Kennerley et al., 2005) and oxygen saturation estimated to be 50% when breathing room air. Changes in haemoglobin oxygenation and saturation were thus calculated on a pixel by pixel basis, before conversion to 3-D datasets (2-D images over time) for HbO_2_, HbR and HbT changes.

### 2.5 Stimulation paradigms

Stimulation of the whisker pad was delivered via two subdermal stainless steel needle electrodes (12 ​mm ​× ​0.3 ​mm, Natus neurology Incorporated, USA) directly inserted into the whisker pad which transmitted an electrical current (1.0 ​mA). This intensity has been shown to evoke a robust haemodynamic response without altering physiological factors such as blood pressure and heart rate. The whiskers were stimulated at one of six frequencies (1, 2, 5, 10, 20 & 40Hz), for 2s with a stimulus pulse width of 0.3ms. The order of stimulation frequencies was pseudorandomised with 10 trials at each frequency and an inter-trial interval (ISI) of 25s. The electrical current is generated by an independent amplifier (Isolated Stimulator DS3, Digitimer Ltd., UK) which directly attaches to the electrodes. All stimulation paradigms were carried out commencing at 4 and 6 ​h after LPS/vehicle administration.

### 2.6 Hypercapnia challenge

A hypercapnia challenge was used as a measure of vascular reactivity, independent of neuronal activity changes. During hypercapnia, a 10% concentration of carbon dioxide in medical air (9L medical air, 1L CO₂) was administered to the air supply tube of the ventilator. Thirty-second long challenges were repeated four times at intervals of 210s in the absence of whisker stimulation. An interval of 210s ensured that the animal’s physiological parameters returned to baseline levels before delivering the next challenge. These challenges were performed following the whisker stimulation paradigm.

### 2.7 Perfusion

Rats (*n* ​= ​11) were transcardially perfused 8 ​h after LPS/vehicle administration (following *in vivo* data collection) with saline (0.9% warmed to 37 ​°C) with the addition of heparin (0.1ml/500 ​ml) to exsanguinate the vessels, and subsequently fixed in 4% paraformaldehyde (PFA) 01.M pH 7.4 in PBS. Saline and fixative were administered through a pump (Masterflex L/S, Cole-Parmer Instrument Company, UK) at a rate of 34 ​ml/hr. Brains were stored in PFA overnight at 4 ​°C, sub-dissected into four regions, and embedded in paraffin wax. Serial sections (5 ​μm) were cut from the paraffin-embedded tissue.

### 2.8 Immunohistochemistry

Immunohistochemistry was performed using a standard avidin-biotin complex-horse radish peroxidase (ABC-HRP) method, and visualised with diaminobenzidine ([DAB], Vector Laboratories, UK). A summary of utilised primary antibodies and their conditions of use are shown in Table 1. Isotype and no primary antibody controls were included in every run and no specific immunoreactivity was observed.

**Table 1:** Antibody sources and experimental conditions.

Antibody	Isotype	Dilution (time, temp)	Antigen retrieval	Supplier
Anti-GFAP	Rabbit IgG	1:1000 (1 ​hr RT)	MW 10 ​min, pH9	Dako, UK
Anti-IBA1	Rabbit IgG	1:400 (1 ​hr RT)	MW 10 ​min, pH9	Abcam, UK
ICAM-1	Goat IgG	1:400 (1 ​hr RT)	MW 10 ​min, TSC pH 6.5	R&D, UK

### 2.9 Data processing and analysis

#### 2.9.1 In vivo imaging data

Data were processed in Matlab (2016a) using custom written code. The same analysis approach was used for the 2D-OIS and LSCI data. Pre-processed (see Section 2.5) 3-D imaging datasets (2-D spatial images, over time) were spatially smoothed and then analysed using SPM (The FIL Methods Group U, 2016), which was implemented through a graphical user interface constructed in Matlab. SPM produces a thresholded activation map, from which regions of interest (ROIs) were selected. Each ROI included contributions from arterial, venous and parenchymal (capillary bed) compartments of the visible cortical surface. Analysis of the 2D-OIS data, which included datasets for concomitant changes in HbO_2_, HbR and HbT, was carried out for the HbT changes and the same ROI was used to extract HbO_2_ and HbR time series. Two animals, one from each group, were excluded from final analysis due to the absence of well-localised stimulus-evoked responses, thereby a total *n* ​= ​14 was used for final analyses (*n* ​= ​7 for each group). ROI size was consistent across animals, for both LSCI (*F*(1,13) = ​0.06, *p* ​= ​.811) and OIS (*F*(1,13) = ​1.54 *p* ​= ​.239) data. LSCI and 2D-OIS time series of haemodynamic changes for each stimulation trials were then extracted from the ROI. LSCI time series were down-sampled to 5Hz (from 25Hz). Data from each stimulation trial were extracted and divided by the pre-stimulus baseline period (10s), to yield a measure of percentage change (fractional changes) in CBF, HbO_2_, HbR and HbT. Time series were averaged across trials according to stimulation condition. Area under the curve (AUC) and maxima for each response were calculated.

Statistical comparisons between groups using maxima and AUC response values were performed using multivariate ANOVAs (MANOVAs) for each time point (4 and 6 ​h). A *p* value below .05 was considered to be a significant effect. Additional independent sample t-tests were conducted to further probe differences between experimental conditions. All statistical analyses were conducted using SPSS 23.

#### 2.9.2 Immunohistochemistry data

All images were taken from the contralateral somatosensory cortex (SS) of the thinned cranial window (right side), to ensure inflammatory effects were not surgery (thinned cranial window) dependent. As a further control, images from the hippocampal CA1 region were taken and quantified. In the SS cortex three adjacent belt transects from the outer cortex through to the white matter border were taken for each animal at x20 magnification (Nikon microscope). SS area coordinates for captured images were taken between −0.40 to −1.80 from Bregma (B) (Paxinos and Watson, 1998). In the hippocampus, random field images were taken for each animal in CA1 region, area coordinates for captured images were between −3.30 to −5.30 from B (Paxinos and Watson, 1998). Percentage GFAP, IBA-1 and ICAM-1 area immunoreactivity was quantified using Analysis^D^ software. All slides were imaged and analysed blind in a randomised order. One vehicle animal was excluded from analysis due to infection. Statistical analyses (independent sample t-tests) were performed using SPSS 23. A *p* value below .05 was deemed significant.

## 3 Results

### 3.1 Acute LPS treatment does not change baseline CBF

To assess any effects of treatment upon baseline CBF, the average perfusion value across a 30s period prior to the onset of stimulation at the start (4 h after LPS or saline administration) and at the end of the experimental protocol (6 h after LPS or saline administration) was calculated for each animal. A mixed design ANOVA on experimental group and time-point revealed no significant main effect of time (4 vs. 6 h; *F*(1,13) = 0.98 *p* = .34) or treatment group (LPS vs. saline; *F*(1,13) = 0.32, *p* = .58) and no significant interaction effect of time and group (*F*(1,13) = 1.02, p = .33). Because laser speckle values are semi-quantitative and may be affected by minor differences in experimental setup between animals, we also calculated the relative change in perfusion baselines between the 4 h and 6 h time-points within each animal. A one-way ANOVA revealed no significant differences in changes in perfusion baseline over this time period between the experimental groups (*F*(1,13) = 0.87, *p* = .37).

### 3.2 Acute LPS treatment alters cerebrovascular responses to whisker stimulation

**Fig. 2:**
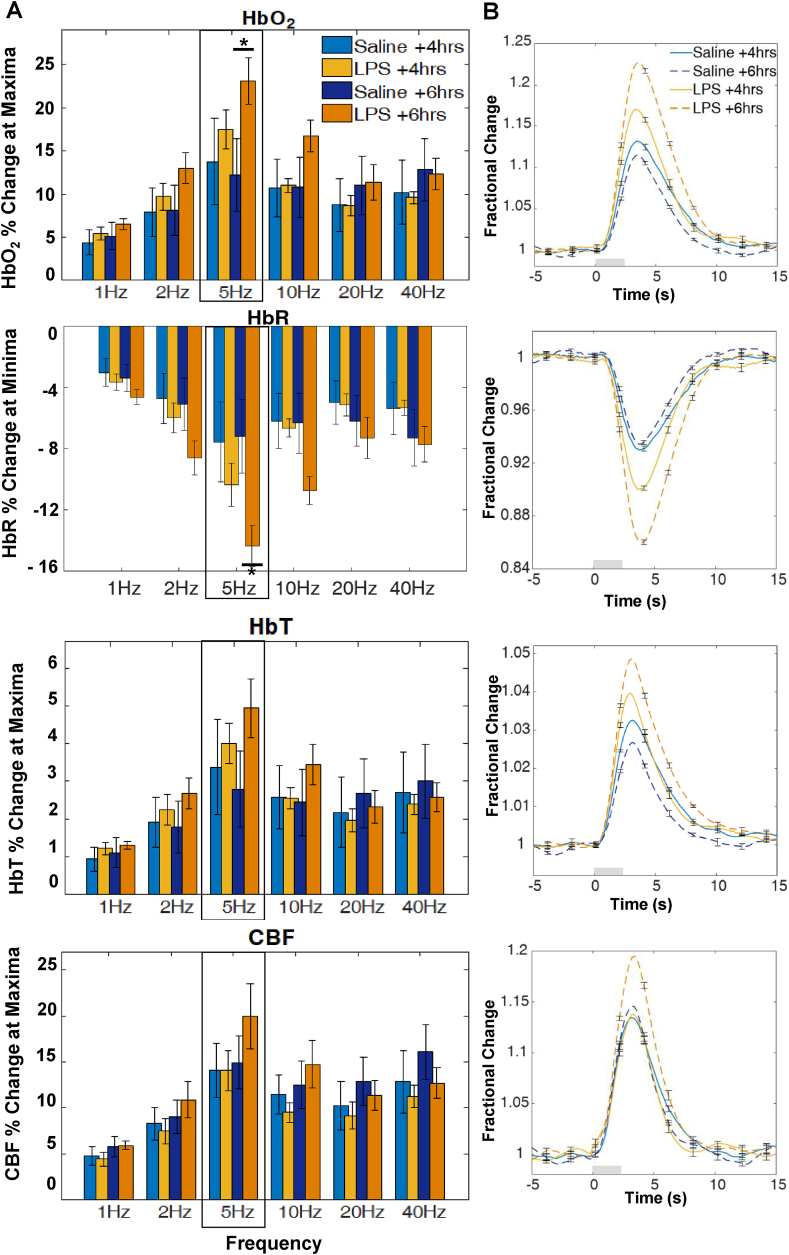
**Haemodynamic response to 2s whisker stimulation at six frequencies at 4 h and ​6 ​h after LPS/saline vehicle administration. (A)** Bar charts show percentage (%) change at maxima or minima (HbR). Post hoc analysis on stimulation frequencies reveal significant effects at 5Hz for HbO_2_ and HbR (∗denotes significant differences at *p* < ​.05) between groups **(B)** Representative 5Hz time series showing mean fractional changes. Grey rectangle indicates stimulation onset/offset and error bars indicate standard error of the mean.

Multivariate analyses of variance (MANOVAs) were applied to HbO_2_, HbR, HbT and CBF response maxima or minima (HbR) values in order to determine significant effects of LPS administration at 4 ​h and at 6 ​h post treatment. All cases (*n* ​= ​14) were included in the analysis. At 6 ​h, LPS administration altered the profile of haemodynamic responses across the investigated stimulation frequency range with a significant interaction between stimulation frequency and (LPS or vehicle) treatment (*F*(20, 190) = ​2.31, *p* ​= ​.002; Wilks’ Λ ​= ​0.486). There were also significant univariate interaction effects for each haemodynamic measure as reported in Table 2. Post hoc analysis of individual stimulation frequencies using independent t-tests (Fig. 2 A) was used to assess if any particular frequency was more salient in driving the above interaction effect. Results indicate a significant increase in HbO_2_ (*p* ​= ​.0496) and HbR (*p* ​= ​.022) response magnitude at 5Hz following LPS treatment. A representative haemodynamic response profile at a 5Hz stimulation frequency is plotted in Fig. 2 B for each measure.

**Table 2:** Summary of univariate statistics for haemodynamic responses to a mixed frequency 2s stimulation paradigm.

Time	Factor		*df*	*F*	*p*
4 ​h after LPS/vehicle administration	Frequency	HbO_2_	5,60	15.19	< .001
		HbR	5,60	18.68	< .001
		HbT	5,60	11.80	< .001
		CBF	5,60	17.98	< .001
	Frequency x Treatment	HbO_2_	5,60	0.76	= ​.581
		HbR	5,60	1.45	= ​.219
		HbT	5,60	0.54	= ​.743
		CBF	5,60	0.23	= ​.949
	Treatment	HbO_2_	1,12	0.10	= ​.758
		HbR	1,12	0.24	= ​.635
		HbT	1,12	0.02	= ​.892
		CBF	1,12	0.15	= ​.703
6 ​h after LPS/vehicle administration	Frequency	HbO_2_	5,60	21.45	< .001
		HbR	5,60	23.86	< .001
		HbT	5,60	15.25	< .001
		CBF	5,60	19.13	< .001
	Frequency x Treatment	HbO_2_	5,60	6.48	< .001
		HbR	5,60	7.92	< .001
		HbT	5,60	4.75	= ​.001
		CBF	5,60	2.64	= ​.032
	Treatment	HbO_2_	1,12	1.21	= ​.294
		HbR	1,12	2.36	= ​.151
		HbT	1,12	0.43	= ​.524
		CBF	1,12	0.07	= ​.798

At 4 ​h post injection, response magnitudes are increased (HBO_2_, HBT, CBF) or decreased (HbR) and are especially evident in the 5Hz stimulation trials (Fig. 2), but this is not supported by a MANOVA, with no significant interaction between stimulation frequency and treatment (*F*(20, 190) ​= ​1.34, *p* ​= ​.156; Wilks’ Λ ​= ​0.645).

At both time-points treatment condition by itself did not result in any significant effect on response magnitudes (4 h: *F*(4,9) = ​0.33, *p* ​= ​.852; Wilks’ Λ ​= ​0.872; 6 h: *F*(4,9) = ​1.25, *p* ​= ​.357; Wilks’ Λ ​= ​0.643). Thus although a consistent trend in increases in haemodynamic response was observed in LPS-treated animals, the interaction with stimulation frequency was key in driving significant differences between groups.

At both time points there was a significant effect of stimulation frequency on haemodynamic response magnitude (4 h: *F*(20, 190) ​= ​7.042, *p* < ​.001; Wilks’ Λ ​= ​0.159; 6 h: *F*(20, 190) ​= ​6.41, *p* < ​.001; Wilks’ Λ ​= ​0.181 [Fig. 2 A]), indicating the range of stimulus inputs was effective in driving responses over a dynamic range, with significant effects for each haemodynamic response measure (Table 2).

### 3.3 LPS administration does not change haemodynamic responses to hypercapnia

**Fig. 3:**
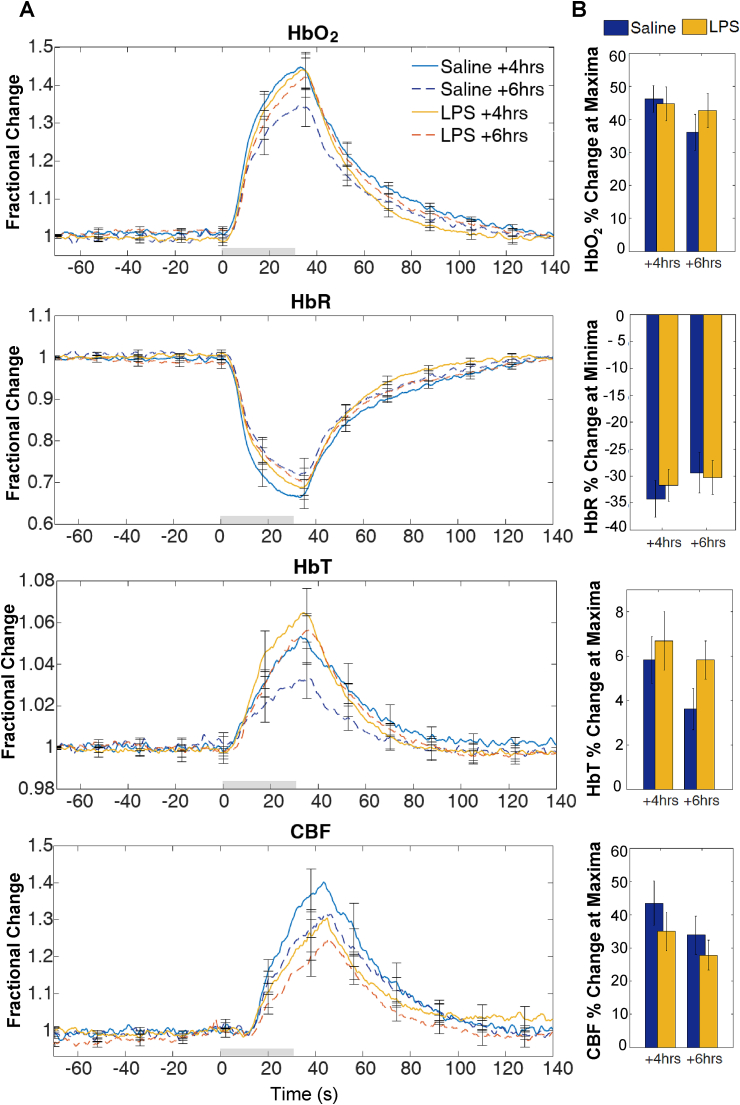
**HbO**_**2**_**HbR, HbT and CBF response to hypercapnia at 4 h and 6 h afterLPS/saline vehicle administration. (A)** Time series show mean fractional changes and **(B)** bar charts show percentage change at maxima or minima. Grey rectangle indicates the 30s CO_2_ challenge onset/offset. Error bars represent standard error of the mean.

Analysis of variance (MANOVA) were used to determine significant effects of treatment on haemodynamic response magnitude (for HbO_2_, HbR, HbT and CBF variables) to a 30s hypercapnia challenge at 4 ​h and 6 ​h post treatment. Maxima (or minima for HbR) values were used to quantify the response. Treatment had no significant effect on response magnitude at 4 ​h (*F*(4,9) = ​1.42, *p* ​= ​.304; Wilks’ Λ ​= ​0.613) or at 6 ​h (*F*(4,9) = ​1.33, *p* ​= ​.331; Wilks’ Λ ​= ​0.629) (Fig. 3).

### 3.4 LPS treated animals show a change in the cerebral metabolic rate of oxygen consumption (CMRO_2_) following treatment

**Fig. 4:**
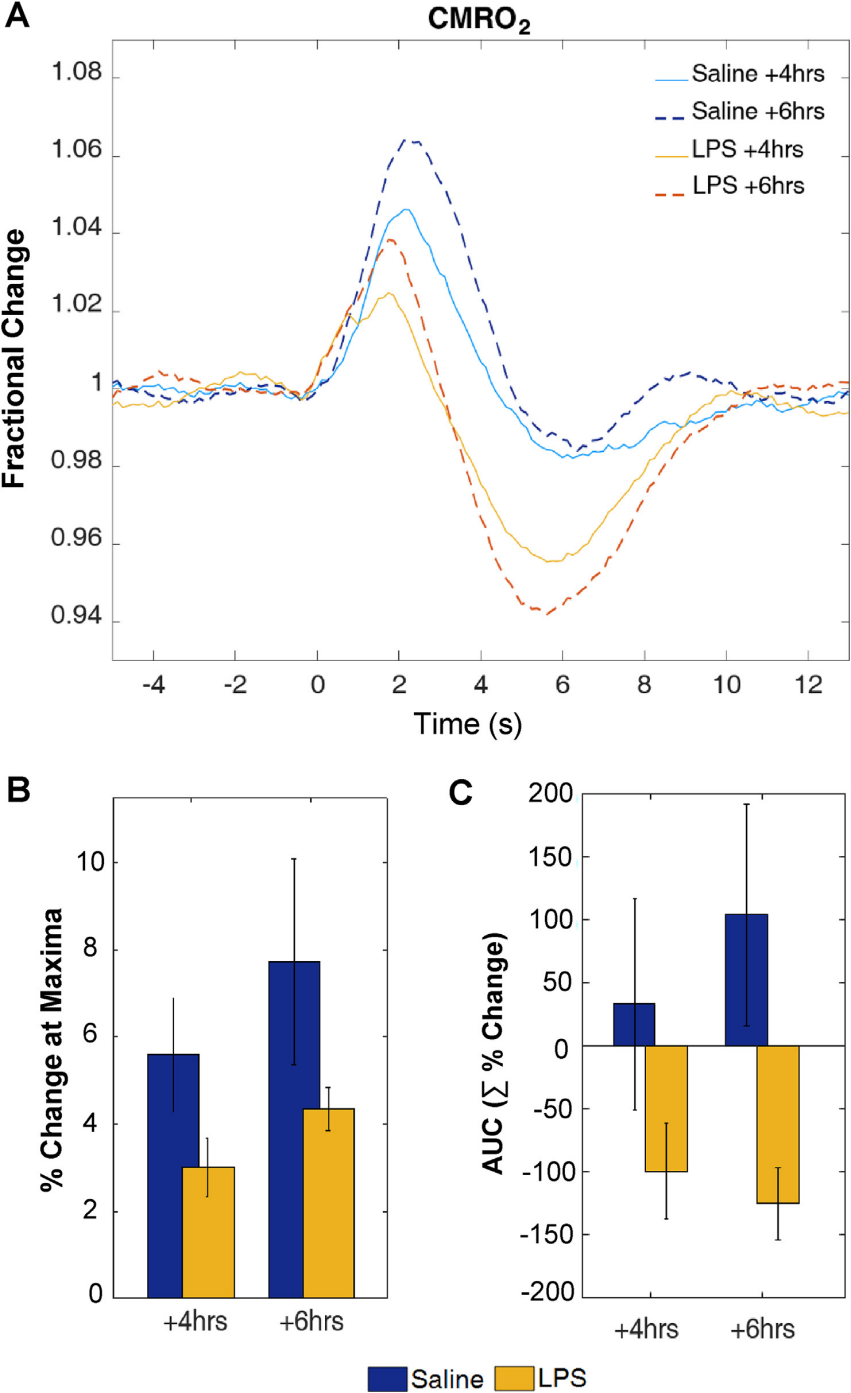
**CMRO**_**2**_**estimation at +4 and +6 after LPS/saline vehicle administration. (A)** Time series of estimated CMRO_2_ changes following whisker stimulation, **(B)** Bar chart showing percentage change at maxima and **(C)** Bar chart showing change in area under the curve (AUC, units are summed percentage change).

The method utilised to estimate CMRO2 was as described in (Jones et al., 2001; Mayhew et al., 2001). Briefly the CMRO_2_ estimate was calculated from HbR, HbT and CBF values generated from 2D-OIS and LSCI data. CMRO_2_ in the brain is directly linked to cellular energy consumption and neuronal activity (Liu et al., 2014), thus it can provide a measure to assess the neurovascular coupling relationship by assessing changes in oxygen delivery or oxygen metabolism. CMRO_2_ changes in response to simulation were estimated at both time points (4 ​h and 6 ​h) and for both vehicle and LPS treated animals (Fig. 4). Response maxima and AUC were calculated and analysed with one-way ANOVA. There was no significant difference in the response maxima between groups at 4 ​h or 6 ​h after LPS/vehicle treatment (4 ​h: *F*(1,13) = ​3.5, *p* ​= ​.087; 6 ​h: *F*(1,13) = ​2.5, *p* ​= ​.138). However, for AUC, a one-way ANOVA reveals a significant difference between treatment groups at 6 ​h (*F*(1,13) = ​6.16, *p ​=* .029). Inspection of the time-series of changes in Fig. 4 indicate that this difference is attributable to a substantial below baseline decrease in CMRO_2_, following an initial increase, in LPS treated animals. No significant difference in AUC was found at 4 ​h (*F*(1,13) ​= ​2.05, *p* ​= ​.178).

### 3.5 Acute LPS treatment induces astrogliosis and microgliosis

**Fig. 5:**
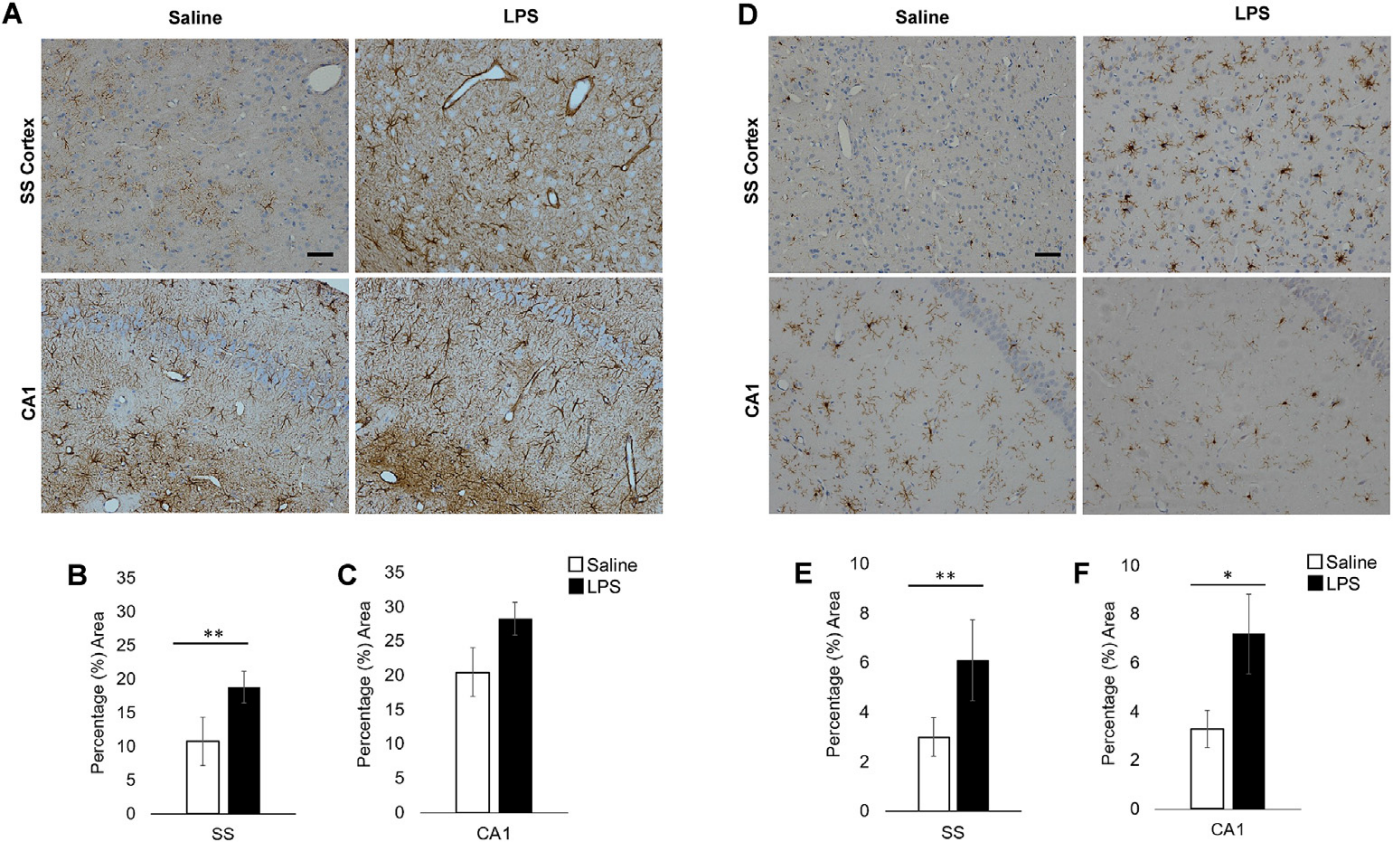
**LPS-treatment changes in glial pathology. (A)** GFAP immunolabelling of astrocyte cell body and immediate processes. **(B)** GFAP SS percentage area immunoreactivity was significantly increased in LPS treated animals (74% increase, *p* ​= ​.003) but was not significantly different in CA1 (*p* ​= ​.089) **(C)**. **(D)** IBA1 immunolabelling of microglia cell body and ramified processes. IBA1 SS **(E)** and CA1 **(F)** percentage area immunoreactivity were significantly increased in LPS treated animals (SS: 109% increase, *p* ​= ​.005; CA1: 119% increase *p* ​= ​.003). Scale bar represents 50 ​μm ∗∗ denotes *p* < ​.010 ∗ denotes *p* < ​.05.

GFAP immunolabelled the cell body and immediate processes of astrocytes in both cohorts (Fig. 5 A). Hypertrophic astrocytes were observed in LPS cases, indicative of a mild to moderate astrogliosis phenotype. Levels of GFAP expression, assessed as percentage area immunoreactivity, showed a significant 74% increase in LPS cases in the SS cortex (*t*(8) = ​−4.15, *p* ​= ​.003) (Fig. 5 B). Within the CA1 region of the hippocampus immunoreactivity was similar to control cases, no significant difference was found between LPS and vehicle treated groups (Fig. 5 C), *t*(8) = ​−1.94, *p* ​= ​.089 [38% increase]).

IBA1 immunolabelled the cell body and ramified processes of microglia in both treatment groups (Fig. 5 D). LPS-treated animals displayed a hypertrophic profile, indicative of a reactive microglial phenotype with a larger, more rounded cell body. Levels of IBA1 expression, assessed as percentage area immunoreactivity, showed a significant 109% increase in LPS treated animals in SS cortex (*t*(8) = ​−3.84, *p* ​= ​.005) (Fig. 5 E) with more intense immunolabelling of both the cell body and extending processes. In the CA1 hippocampal region, IBA1⁺ microglia immunoreactivity for LPS treated was also significantly increased (*t*(8) = ​−4.09 [119% increase], *p* ​= ​.003, Fig. 5 F).

### 3.6 Acute LPS treatment increased expression of ICAM-1 on the endothelial luminal surface and microglia processes

**Fig. 6:**
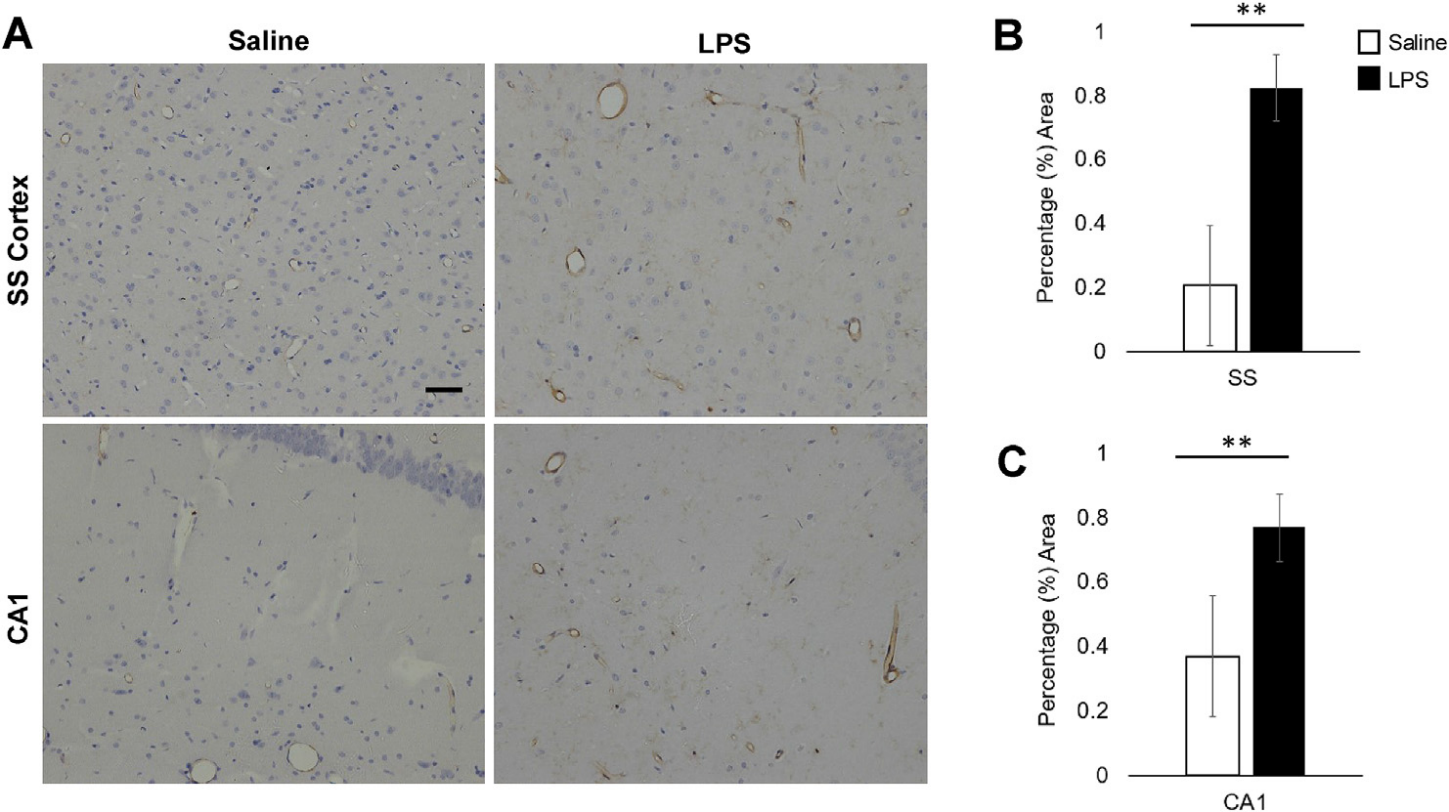
**LPS-treatment changes in ICAM-1 expression. (A)** ICAM-1 immunolabelled vessels and microglia processes. LPS-treatment led to increased percentage area ICAM-1 immunoreactivity in the SS cortex **(B)** (299% increase, *p* < ​.001) and in the CA1 **(C)** (108% increase, *p* ​= ​.001). Scale bar represents 50 ​μm ∗∗ denotes *p* < ​.002.

ICAM-1 immunolabelled vessels in both cohort groups although LPS treated animals showed more intense immunoreactivity compared to controls (Fig. 6 A). Furthermore, immunolabelling of microglia was a feature of all LPS cases but was less intense than vessel labelling. Percentage area assessment revealed a significant increase in ICAM-1 expression in LPS cases with a 299% area increase in the SS cortex (*t*(8) = ​−6.5, *p* < ​.001, Fig. 6 B) and 108% area increase in CA1 (*t*(8) ​= ​−4.73, *p* ​= ​.001, Fig. 6 C).

## 4 Discussion

In this study we present evidence of acute alteration of cerebrovascular function by systemic inflammation, using an animal model treated with LPS. This was demonstrated by changes in cortical haemodynamic responses to stimulation, an absence of effects on baseline blood flow or vascular reactivity, and concomitant cellular changes in the NGVU. Although direct comparisons of responses to various inflammatory stimuli between species are difficult, these findings may have implications for the interpretation of functional neuroimaging data acquired in clinical and healthy human cohorts; the presence of systemic inflammation may change the haemodynamic response processes which underlie neuroimaging signals (e.g. CBF, cerebral blood volume (CBV) and CMRO_2_). In addition, characterisation of NGVU cellular changes via immunohistochemistry showed morphological changes in astrocytes and microglia, alongside a marked increase in ICAM-1 expression, indicating EC activation.

LPS administration significantly changed cerebrovascular function as indicated by an interaction of stimulation frequency (input to the cortex) and treatment condition. These effects became evident at 6 ​h after LPS administration, although similar (non-significant) trends were observed at the 4 h time point. The significant effects on cerebrovascular function were mainly manifested as an increased response at a specific stimulation frequency (5Hz), although similar trends were indicated at other frequencies. Stimulation frequency dependent effects have been previously reported in the literature (Spain et al., 2015; Martin et al., 2006) and for the whisker system, stimulation frequencies at around 5Hz are regarded as most effective and of functional relevance in driving somatosensory neurovascular responses in anaesthetised animals (Martin et al., 2006; Adibi, 2019) and may therefore represent the point of maximal sensitivity to treatment effects in this study. These results underline the complexity of the neurovascular coupling relationships, and the importance of including a dynamic range of sensory stimulation in neurovascular research to better assess how the neurovascular system responds to different levels of input in health and under disease-related manipulations.

Hypercapnia is a widely used protocol, in both animal (Martin et al., 2006) and human (Suri et al., 2015) research studies, for assessing and comparing cerebrovascular reactivity. Here, we report that the cerebrovascular responses to hypercapnia are not significantly changed by LPS administration. This, together with a lack of evidence of altered baseline CBF, indicates that the changes in haemodynamic responses to stimulation are thus not a consequence of a generic effect on vascular reactivity or baseline blood flow, but instead may relate to the function of the NGVU in translating changes in neural activity into vascular responses, i.e., neurovascular coupling.

CMRO_2_ in the brain is considered to be an index of brain health and energy homeostasis (Liu et al., 2014) as well as being directly linked to cell energy and neuronal activity (Ge et al., 2012). Estimation of CMRO_2_ can thus provide a measure to assess the neurovascular dynamics by assessing possible changes in oxygen delivery or oxidative metabolism. Our CMRO_2_ estimates suggest a change in how oxygen delivery is matched to metabolic demand; where the magnitude of changes in CBF relative to oxygen consumption is increased by LPS treatment. This may in turn reflect a change in neurovascular coupling but this requires validation with a direct measure of neuronal activity. Our CMRO_2_ estimates indicate a change in the relationship between the HbR, HbT and CBF components of the haemodynamic response under LPS treatment, from which the CMRO_2_ estimate is made. This would in turn predict that BOLD fMRI signals, which are also derived from HbR, HbT and CBF changes, will be different under LPS treatment. Therefore, caution should be used in interpreting neuroimaging signals acquired from subjects with systemic inflammation present.

GFAP⁺ astrocytes in LPS-treated animals displayed significantly greater immunoreactivity of processes and cell bodies, a phenotype suggestive of mild to moderate astrogliosis (Sofroniew and Vinters, 2010). A non-significant difference between GFAP^+^ immunoreactivity in the CA1 region of LPS treated and control animals may be explained by the dense population of GFAP^+^ astrocytes in the hippocampus compared to cortical areas (Gomi et al., 1995; Shibuki et al., 1996), leading to a high level of baseline immunoreactivity and reduced sensitivity to group differences. Further quantification utilising other GFAP isoforms alongside additional astrocyte markers (such as ALDH1L1, nestin, vimentin) may help to fully elucidate the astrocyte response triggered by an acute LPS challenge. Similarly, increases in IBA1 expression and a shift from a ramified ‘resting’ morphology to a hypertrophic profile (characterised by an amoeboid appearance) indicates microgliosis in our LPS treated animals (Boche et al., 2013; Sun et al., 2008).

ICAM-1 has an important role in cell-to-cell adhesion interactions and has low expression in cerebral microvessels in normal physiological conditions (Wertheimer et al., 1992), but is upregulated on the luminal surface of ECs in the presence of pro-inflammatory mediators (Huber et al., 2006; da Fonseca et al., 2014). In LPS treated animals, we demonstrate increased ICAM-1 expression on the luminal surface of ECs, as well as microglial processes. ECs have already been shown to change their phenotypes in support of various phases of the inflammatory process (Pober and Sessa, 2007; Pober and Cotran, 1990). Furthermore, EC location at the interface between brain and blood, as well as their expression of TLR-4 receptors (Verma et al., 2006) (specifically activated by the LPS strain we used), might indicate these cells as initiators of the inflammatory response in this study. Alterations in EC function could then propagate to other NGVU cells, activating astrocytes and microglia. In support of this, a paper developing a concurrent cell-type specific isolation method (Swartzlander et al., 2018), reported gene expression changes (upregulation of pro-inflammatory cytokines, chemokines, cell adhesion molecules, including ICAM1) in vascular endothelia in response to a peripheral LPS challenge. These results indicate that vascular ECs may be central in the initiation and transmission of the LPS response from the periphery to the CNS via cytokines, chemokines and extracellular remodelling (Pober and Cotran, 1990).

ECs have also been implicated as key players in mediating neurovascular coupling in health (Chen et al., 2014). Retrograde dilation of pial vessels following sensory stimulation is blocked if EC signalling is interrupted and disruption of ECs at the pial surface leads to a significant attenuation of the haemodynamic response (Chen et al., 2014). This could explain the increases in haemodynamic response observed in this study, where such changes could be mediated by increased activity of ECs at the pial surface.

CNS resident cells including microglia (Marzolo et al., 2000) and astrocytes (Chakravarty and Herkenham, 2005) possess TLR-4 receptors and thus could be directly activated by an LPS challenge, a possibility that must be considered in the interpretation of our data. However, lipopolysaccharides and pro-inflammatory molecules are large and thus should have limited BBB permeability (Holmes, 2013). Evidence from studies using radioactively labelled LPS (Banks et al., 2015; Banks and Robinson, 2010) has shown little LPS penetration of the BBB (0.025% of the administered dose) and only at doses of 3 ​mg/kg or higher. As such, in our model, and owing to the acute nature of the study, we do not anticipate BBB breakdown and extravasation of LPS into the parenchyma to be a major factor. This suggests that the observable brain inflammatory response produced by a peripheral administration of LPS is most likely mediated and initiated by ECs. It may also be mediated by alternative routes of communication between the brain and the periphery as opposed to a direct effect on glial TLR-4 brain receptors (for a comprehensive review see Holmes, 2013). This could explain why studies comparing brain and peripheral inflammatory challenges such as the one by Montacute et al. (2017) report similar levels of brain inflammation in both challenges.

There is a pressing need to understand the communication pathways between the peripheral and central immune systems in order to understand the role of inflammation in neurological diseases and ageing. Neurodegenerative diseases such as AD are characterised by a chronic low-grade inflammatory response and therefore, a limitation of this study is the use of an acute experimental design. However, because chronic and acute inflammatory processes overlap substantially and share some of the same mediators, including EC activation (Pober and Sessa, 2007), the effects observed here may still be informative when considering the chronic case. Relatedly, ageing is also a key factor in both disease pathology and in inflammation but is not investigated in the present study. As such, future work should aim to extend this model to include low-dose chronic inflammation and the use of aged animals to maximise the relevance of findings to human pathology and human ageing.

Whilst we find no evidence of leukocyte extravasation in LPS treated cases, astrocytes and microglia are highly likely to be contributing to the neuroinflammatory response by secreting an array of cytokines. Future work should thus also aim to characterise the inflammatory profile of glial cells in response to acute systemic inflammation. Lastly, different species of LPS elicit different cytokines profiles, thereby producing different classes of immune response *in vivo* (Pulendran et al., 2001)*.* This is an important consideration for studies utilising LPS to create an experimental model of inflammation. The LPS strain utilised in this study, due to its ultrapure nature, only activates the TLR-4 pathway and thus offers a robust way of investigating the inflammatory-driven neurovascular and NGVU effects which are mediated by a specific pathway.

## 5 Implications and conclusion

This study has implications for the understanding of how cerebrovascular function changes during in response to acute systemic inflammation. The results support our overall hypothesis that a systemic inflammatory challenge may impact upon cerebrovascular function through a shift in the operation of NGVU cells, from neurovascular regulation to neuroinflammatory response. Changes in cerebrovascular function and the coupling of local blood flow to fluctuations in neuronal activity may be of particular importance for human fMRI research studies which rely on assumptions regarding the short-latency impulse response function (as measured here by our stimulation protocol) to make inferences about neuronal activity changes from neuroimaging signals (Logothetis, 2008). Although inflammatory stimuli doses and subsequent response are not straightforwardly comparable between species, the findings from the current study may have implications for the application of fMRI in subjects or patients where a systemic inflammatory response may be present.

In conclusion, our data shows that an acutely induced systemic inflammatory response is able to rapidly change *in* *vivo* cerebrovascular function, with an associated marked immunoreactivity within the cellular constituents of the NGVU. We hypothesise that the inflammatory response may be triggered initially by ECs, as these cells are directly exposed to the bloodstream and have been implicated in mediating neurovascular coupling in health.

## Declarations of competing interest

None.

## List of abbreviations

2D-OIS: Two-dimensional optical imaging spectroscopy
Aβ: Amyloid-beta
AD: Alzheimer’s disease
AQP4: Aquaporin 4
BBB: Blood brain barrier
BOLD: Blood oxygen level dependence
CBF: Cerebral blood flow
CBV: Cerebral blood volume
CMRO_2_: Cerebral metabolic rate of oxygen consumption
DAB: 3,3′-Diaminobenzidine;
fMRI: functional magnetic resonance imaging
GFAP: Glial fibrillary acidic protein
HbO_2_: Oxyhaemoglobin
HbR: Deoxyhaemoglobin
HbT: Total haemoglobin
IBA1: Ionized calcium binding adaptor molecule 1
ICAM-1: Intercellular adhesion molecule
IL-1: Interleukin 1
LPS: Lipopolysaccharide
LSCI: Laser speckle contrast imaging
NIRS: Near infra-red spectroscopy
NGVU: Neuro-glial-vascular unit
VCAM-1: vascular cellular adhesion molecule
PET: Positron emission tomography
SS: Somatosensory cortex
TLR-4: Toll-like receptor 4
TNF-α: Tumour necrosis factor alpha

## Data statement

The datasets used and/or analysed during the current study are available from the corresponding author on reasonable request.
